# Air Quality Risks in Public Housing

**DOI:** 10.1001/jamahealthforum.2026.1357

**Published:** 2026-05-29

**Authors:** Brynn E. Sheehan, Carolyn Caraballo Velez, Vaughan W. Rees, Miasha T. O’Neal, Kristen E. Moore, Katie M. Jones, Jessica Meadows, Sarah Gehlert, Richard Grucza, Andrew Plunk, Kelli J. England

**Affiliations:** 1 Department of Psychiatry and Behavioral Sciences, Virginia Health Sciences at Old Dominion University, Norfolk; 2 Research and Infrastructure Service Enterprise, Virginia Health Sciences at Old Dominion University, Norfolk; 3 Department of Pediatrics, Virginia Health Sciences at Old Dominion University, Norfolk; 4 Department of Social and Behavioral Sciences, Harvard T.H. Chan School of Public Health, Boston, Massachusetts; 5 The Brown School, Washington University, St Louis, Missouri; 6 Departments of Family and Community Medicine and Health and Clinical Outcomes Research, St Louis University School of Medicine, Missouri; 7 Black Mountain, North Carolina

## Abstract

**Importance:**

Public housing residents face elevated environmental health risks, yet indoor air quality in these settings remains underexamined.

**Objective:**

To evaluate indoor air quality in public housing 5 years after implementation of the federal smoke-free housing policy and compare it with outdoor air quality in the same regions.

**Design, Setting, and Participants:**

A 1-year cohort study of indoor air quality (particulate matter ≤2.5 μm in diameter [PM_2.5_]) across 12 multifamily public housing communities in southeastern Virgina for individuals 65 years and older or persons with disability in cities with the highest rated outdoor air quality. Indoor PM_2.5_ was continuously measured using 117 air quality monitors in shared common spaces and hallways of buildings. Outdoor data were obtained from 3 nearby Environmental Protection Agency (EPA) monitoring stations. Data were collected from January 1 to December 31, 2023.

**Exposure:**

Federal smoke-free housing policy implemented in 2018 aimed at reducing indoor pollution from tobacco smoke.

**Main Outcomes and Measures:**

Daily PM_2.5_ concentrations indoors and outdoors classified using EPA-defined risk categories. A repeated-measures linear mixed model was used to assess daily concentration differences over the 1-year study timeframe.

**Results:**

In this study of 12 public housing communities, indoor PM_2.5_ levels were significantly higher than outdoor levels (mean indoor PM_2.5_, 23.33 μg/m^3^; 95% CI, 21.85-24.81 μg/m^3^; mean outdoor PM_2.5_, 8.42 μg/m^3^; 95% CI, 5.49-11.35 μg/m^3^; *P < *.001), with 95% of daily indoor readings in the moderate (PM_2.5_, 9.1-35.4 μg/m^3^) or worse EPA risk categories, while 69% of outdoor readings were classified as good (PM_2.5_, 0-9 μg/m^3^).

**Conclusions and Relevance:**

Results of this study suggest that, despite a strong outdoor air quality rating and a smoke-free housing policy, indoor air quality in public housing remains poor. The findings underscore the need for strong, multifaceted interventions to reduce indoor pollution and promote environmental health equity.

## Key Points

**Question:**

How does indoor air quality in public housing compare with outdoor air quality in a region with an A rating from the Environmental Protection Agency (EPA)?

**Findings:**

In this cohort study of 12 public housing communities in southeastern Virgina, daily indoor PM_2.5_ concentrations were significantly higher than outdoor air concentrations. Most indoor readings were in the moderate or worse EPA categories, while most outdoor readings were classified as good.

**Meaning:**

Results of this study suggest that, even in regions with top-rated outdoor air quality, public housing residents may experience poor indoor conditions, highlighting the need for targeted pollution-reduction and equity-focused interventions.

## Teaser

This cohort study reports on indoor air quality in public housing in a region with top-rated outdoor air quality and a federal smoke-free housing policy.

## Introduction

The Air Quality Index (AQI), adopted by the Environmental Protection Agency (EPA), provides a standardized, daily measure of air pollution based on key pollutants such as ozone and fine particulate matter (particulate matter ≤2.5 μm in diameter [PM_2.5_]). AQI data inform both rapid public health communication and long-term air quality assessments, including the American Lung Association’s State of the Air report, which grades regions based on the number of days they exceed health-based pollution thresholds.^1^

Despite progress in reducing air pollution, the 2024 State of the Air report indicates that approximately 131 million people in the US (or 39% of the population) live in areas that receive failing grades for ozone or fine-particle pollution.^1^ These poor air quality patterns are not evenly distributed. Lower-income and historically marginalized communities are more likely to live near high-pollution areas, contributing to a disproportionately high health burden.^2^

Public housing residents are particularly vulnerable to environmental hazards due to higher smoking rates and secondhand smoke exposure that increase cancer risks and exacerbate preexisting health conditions such as asthma and cardiovascular disease.^3^ External contaminants from nearby industrial sites, railroads, shipyards, and vehicle emissions also disproportionately affect public housing residents. Socioenvironmental stressors, including substandard housing conditions and limited access to quality health care, further compound health risks.^4,5^ Indoor air quality can be worse than outdoor air due to poor ventilation and the buildup of pollutants from mold, cooking, smoking, and building materials.^6^ This poses a substantial concern for public housing residents, who already experience a disproportionate health burden.^7^

In 2018, the US Department of Housing and Urban Development (HUD) implemented a smoke-free housing (SFH) policy to improve indoor air quality in public housing.^8^ However, a previous study by some authors of the present study identified an unintended consequence: indoor air quality worsened, likely due to residents smoking indoors to avoid detection and citation.^9^ Despite the intention of the HUD indoor smoking ban to reduce secondhand smoke exposure and tobacco-related harms, poor rule compliance in a setting chronically beset with problems of poor air quality may have resulted in little improvement in air quality to benefit residents’ health.^9,10^

Hampton Roads, a 7-city coastal region of southeastern Virginia, has earned an A outdoor AQI rating, reflecting healthy air conditions in the region.^1^ However, this rating fails to account for disparities across subpopulations or subregions, for example, high-risk environments such as income-based public housing. To determine whether the SFH policy has effectively improved indoor air conditions, we examined indoor air quality in income-based public housing and compared it with outdoor air quality in the same region, 5 years after the adoption of the SFH policy.

## Methods

**Figure 1.**
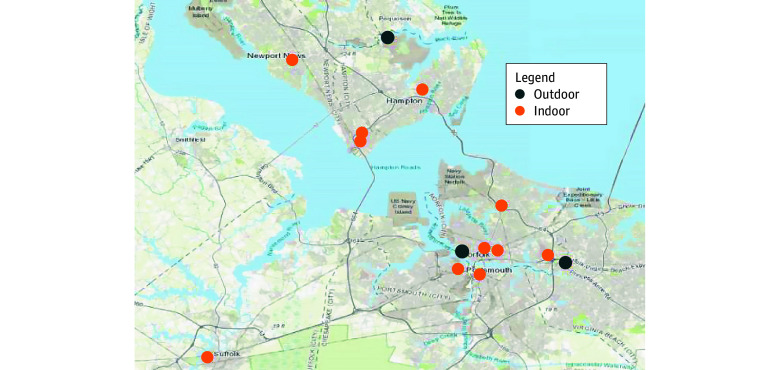
Geographic Map of Hampton Roads, Virginia Air Quality Monitor Locations Data were visualized using ArcGIS Pro version 3.30 (Esri).

Indoor air quality was assessed via daily measurement of indoor particulate matter (PM_2.5_) using 117 air quality sensors (PurpleAir LLC), placed in 12 mid- and high-rise public housing apartment buildings for individuals 65 years and older or persons with disability in Hampton Roads, Virginia.^11^ Property sizes ranged from 47 to 140 units (eTable in Supplement 1 presents indoor site descriptions). Monitors were placed in shared indoor spaces including hallway common areas on floors and within each building wing. Locations were chosen to maximize comparability between buildings. Data were collected from January 1 to December 31, 2023. Indoor air quality data collection was approved by Eastern Virginia Medical School of Old Dominion University Institutional Review Board in Norfolk, Virginia. EPA-reported daily outdoor PM_2.5_ data were collected from 3 Hampton Roads monitoring locations during the same timeframe (Figure 1). We examined the frequency with which daily average PM_2.5_ concentrations were within the EPA descriptor ranges: good (PM_2.5_, 0-9 μg/m^3^), moderate (PM_2.5_, 9.1-35.4 μg/m^3^), unhealthy for sensitive groups (PM_2.5_, 35.5-55.4 μg/m^3^), unhealthy (PM_2.5_, 55.5-150.4 μg/m^3^), very unhealthy (PM_2.5_, 150.5-250.4 μg/m^3^), and hazardous (PM_2.5_>225.5 μg/m^3^).^12^ We followed the Strengthening the Reporting of Observational Studies in Epidemiology (STROBE) reporting guideline for reporting observational studies to ensure transparency and completeness.

### Statistical Analysis

A mixed model was used to estimate means and appropriate CIs for repeated measurements, and differences between indoor and outdoor PM_2.5_ were assessed daily over the 1-year timeframe. Statistical significance was set at 2-sided *P* < .05. Analyses were conducted using IBM SPSS Statistics, version 31.0 (IBM Corporation).

## Results

**Figure 2.**
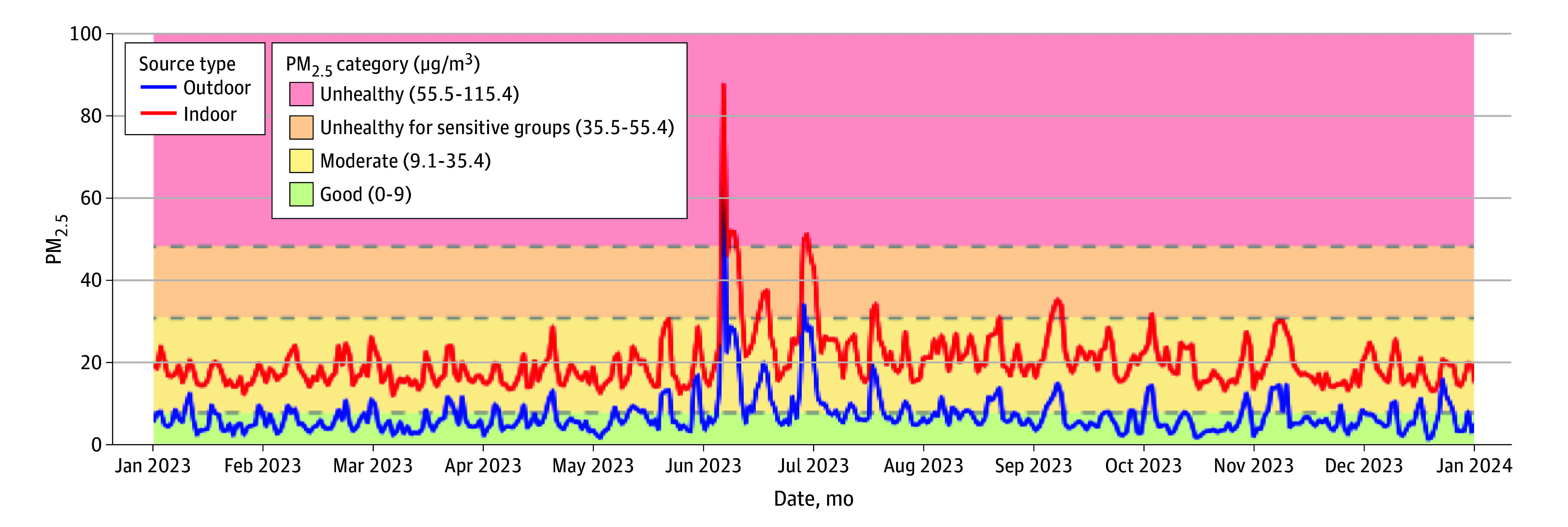
Line Graph Showing a Comparison of Indoor and Outdoor PM_2.5_ Concentrations Over Time PM_2.5_ indicates particulate matter less than or equal to 2.5 μm in diameter.

In this cohort study of 12 public housing communities in southeastern Virgina, indoor PM_2.5_ values ranged from 0.63 μg/m^3^ to 148.36 μg/m^3^ (mean [SD], 23.42 [15.98] μg/m^3^); nearly 95% of daily values were in the moderate range for air quality, 4% were considered unhealthy for sensitive groups, and just over 1% were unhealthy. In contrast, outdoor PM_2.5_ values ranged from 1.40 μg/m^3^ to 88.40 μg/m^3^ (mean [SD], 8.62 [6.35] μg/m^3^); 69% of daily values were considered good, 31% were moderate, and less than 1% were unhealthy for sensitive groups and unhealthy groups (Figure 2). Mean PM_2.5_ levels were significantly worse than outdoor levels (mean indoor PM_2.5_, 23.33 μg/m^3^; 95% CI, 21.85-24.81 μg/m^3^; mean outdoor PM_2.5_, 8.42 μg/m^3^; 95% CI, 5.49-11.35 μg/m^3^; *P* < .001).

## Discussion

We found indoor air quality in public housing communities in eastern Virginia to be significantly worse than outdoor air quality, despite the region’s A rating for outdoor air quality and the adoption of an indoor smoking ban in 2018. The pronounced increase in PM_2.5_ concentrations observed in June 2023 corresponded to the Canadian wildfire smoke that reached the region (Figure 2) and prompted a Code Orange air quality alert, indicating conditions unhealthy for sensitive groups (eg, children and older adults).^13^ Concurrent increases in both outdoor EPA-reported and indoor PM_2.5_ levels support the validity of the indoor air quality measures. Notably, although public health guidance recommends remaining indoors during poor outdoor air quality events, our data show that indoor air quality in public housing for individuals 65 years and older or persons with disability remained consistently worse throughout the year, including during the wildfire period, underscoring a persistent environmental health inequity.

Elevated indoor PM_2.5_ levels pose questions about the general quality of the environmental health of public housing communities and reinforce concerns about the effectiveness of the indoor smoking ban. A 2020 study reported that average indoor PM_2.5_ values initially dropped from 27.48 μg/m^3^ to 19.94 μg/m^3^ 1 month after the SFH policy took effect but returned to 25.18 μg/m^3^ after 1 year.^9^ Current findings confirm the persistence of this issue, with average indoor PM_2.5_ levels over a 12-month period measuring 23.33 μg/m^3^, nearly 3 times higher than outdoor levels of 8.42 μg/m^3^. Our findings suggest that initial air quality improvements that resulted from the adoption of the SFH policy have not been sustained, highlighting the need for longer-term SFH policy implementation and enforcement strategies in public housing communities.

Research suggests that the HUD smoke-free policy has been implemented inconsistently and that communication surrounding the policy is suboptimal, contributing to a lack of clarity regarding what is permitted.^9^ This confusion has increased following the state of Virginia’s legalization of cannabis in 2021. Barriers to policy compliance also should be addressed, including residents’ fear for their safety when stepping off public housing property to smoke. A historical legacy of systemic racism has positioned public housing developments near environmental hazards such as railroads and shipyards, further contributing to exposure disparities.^14^ With this in mind, we selected outdoor air monitors supported by the EPA that are also situated in proximity to these environmental conditions, yet indoor air quality in public housing still proved to be significantly worse than the outdoor air quality.

Our findings reveal substantially higher indoor PM_2.5_ levels indoors compared with outdoors in public housing, a pattern that differs from prior studies of predominantly market-rate US residences that generally reported lower indoor than outdoor PM_2.5_ concentrations and mean indoor levels near 3 to 4 μg/m^3^.^15^ In those settings, higher PM_2.5_ levels are typically episodic and associated with cooking (1.4-23 μg/m^3^) and smoking (15 μg/m^3^), with higher concentrations in multifamily compared with single-family housing.^15,16^ Our findings suggest that indoor smoking functions as a persistent indoor emission source in multiunit, income-based housing, negating the protective effect of remaining indoors during periods of poor outdoor air quality.

### Limitations

Limitations include the use of regional EPA outdoor air quality monitors rather than one-to-one colocated outdoor monitors for each indoor site, which limits building-specific outdoor-to-indoor comparisons but provides the only EPA-validated outdoor reference data available for the study region. In addition, airborne nicotine was not measured, preventing direct confirmation that elevated indoor PM_2.5_ was attributable to tobacco smoke, although findings align with prior evidence of persistent indoor smoking and smoke-free housing policy implementation challenges in public housing.

## Conclusions

Results of this cohort study suggest that a critical reassessment of efforts to fully implement the SFH policy is needed to optimize its overall impact on air quality and health outcomes. Additional support for engaging residents on health education to reduce indoor pollutants, guidance on how to adhere to the SFH rule, and smoking cessation support, while incorporating mitigation strategies such as improved ventilation and air filtration systems, are essential to protecting residents’ health. Policymakers and housing authorities must consider a multifaceted approach to improving indoor air quality, extending beyond smoking bans to address structural and environmental determinants of health. Addressing these disparities is crucial to ensuring equitable environmental health protections for all residents of income-based public and affordable housing communities.

## Notes

Supplement 1. **eTable.** Public Housing Sample Characteristics: Midrise and Highrise Sites in Hampton Roads, VA

Supplement 2.
Data Sharing Statement
