# Supplementary material for: Air Quality Risks in Public Housing
Source: JAMA Health Forum. 2026 May 29;7(5):e261357. doi: 10.1001/jamahealthforum.2026.1357 (PMC13221683; doi:10.1001/jamahealthforum.2026.1357)
Supplement: Supplement 1. — eTable. Public Housing Sample Characteristics: Midrise and Highrise Sites in Hampton Roads, VA [file jamahealthforum-e261357-s001.pdf]

Supplemental Online Content

Sheehan B, Caraballo Velez C, Rees V, et al. Air quality risks in public housing. *JAMA Health Forum*. 2026;7(5):e261357. doi:10.1001/jamahealthforum.2026.1357

eTable. Public Housing Sample Characteristics: Midrise and Highrise Sites in Hampton Roads, VA

This supplemental material has been provided by the authors to give readers additional information about their work.

© 2026 Sheehan B et al. *JAMA Health Forum*.

eTable. Public Housing Sample Characteristics: Midrise and Highrise Sites in Hampton Roads, VA

Characteristics	Number of Sites
**Overview**	
Total Sites	12 (47-140 units per site)
**Property Type**	
Multifamily Senior/Disabled Midrise	10
Multifamily Senior/Disabled Highrise	2
**Range Type**	
Electric	10
Gas	0
Gas and Electric	2
**Air Conditioning Type**	
AC Window Unit	9
Central Air	3
**Pets Allowed**	
Yes	10
No	2
**Hood Fan Presence**	
Yes	5
No	7

© 2026 Sheehan B et al. *JAMA Health Forum*.
