# Supplementary material for: Air Quality Risks in Public Housing
Source: JAMA Health Forum. 2026 May 29;7(5):e261357. doi: 10.1001/jamahealthforum.2026.1357 (PMC13221683; doi:10.1001/jamahealthforum.2026.1357)
Supplement: Supplement 2. — Data Sharing Statement [file jamahealthforum-e261357-s002.pdf]

Data Sharing Statement

Sheehan. Air Quality Risks in Public Housing. *JAMA Health Forum*. Published May 29, 2026.
doi:10.1001/jamahealthforum.2026.1357

Data
Data available: No

Additional Information
Explanation for why data not available: The data that support the findings of this study are available from the corresponding author upon reasonable request.
